# Gleanings in Science and Medicine

**Published:** 1893-04-01

**Authors:** 


### Gleanings in Science and Medicine.

The American Philological Association have a great deal to say regarding our English spelling. Amongst other denunciatory assertions on this subject, our transatlantic critics affirm ours to be "the worst spelling in the world." A code of changes, which they would wish to see brought about, and which Congress has been asked to insist on, in all official printing we quote below: (1) Drop *ue* at the end of words like dialogue, catalogue, &c., where the preceding vowel is short; thus spell demagog, epilog, synagog, &c. (2) Drop final *e* in such words as definite, infinite, favourite, &c., where the preceding vowel is short; thus spell opposit, preterit, hypocrit, requisit, &c. (3) Drop final *te* in words like quartette, coquette, cigarette, &c.; thus spell cigaret, roset, epaulet, &c. (4) Drop final *me* in words like programme; thus spell program, oriflam, &c. (5) Change *ph* to *f* in words like phantom, telegraph, phase, &c.; thus spell filosofer, fonetic, fotografer, paragraf. (6) Substitute *e* for the diphthongs *æ* and *œ* when they have the sound of that letter; thus spell esthetic, eolian, subpena atheneum, &c.

The importance of cremation, as opposed to ordinary interment, has again been brought before the public in a prominent way through the medium of the influential deputation which waited lately on the Home Secretary. Sir Henry Thompson, whose name is well known in the Burial Reform question, was amongst those who interceded on this occasion. It is on the Infectious Diseases question that the promoters of cremation have taken up their line of argument. If, they ask, churchyards are not unhealthy, why then have these burial grounds been transferred from town centres to less frequented parts? England and Wales registers yearly more than half a-million deaths, 70,000 of which have been estimated to arise from causes of infectious disease. With the present accepted form of disposing of the dead, the seeds of contagion are spread broadcast upon the community; indeed, to quote an instance in illustration of this fact, Sir Henry Thompson referred to the last general visitation of cholera in England, when the disease was observed to assume a more virulent form in districts bordering on ground in which the bodies of victims to this disease had been consigned. The germs of contagious diseases are not destroyed through the earth to earth process of disposing of the dead, and the promoters of cremation declare that sooner or later the old order will give way to the new, and that questions of sanitation will supersede questions of sentiment.

The use of alcoholic stimulants is not confined to man; horses, now, it would appear, must be also enrolled amongst the great band of the intemperate. Colonel Spohr, writing in a German military newspaper, points out how frequent is the case in his country of horse trainers forcing their animals to indulge in alcoholic "pick-me-ups." The writer in this article is not inveighing against the dumb animals themselves, nor does the Colonel inform us whether they on their part take kindly to this treatment; but, speaking from many years' experience on the subject, he declares that the effect of alcohol on horses is of a highly injurious nature, and though the heart's action is momentarily accelerated, this is followed by a corresponding period of exhaustion and inaction.

Young Oxford has had a good deal to say lately in a maledictory manner against the threatened innovation of indiarubber shoes for proctorial use, as also for the use of their satellites, the Bull dogs, of University fame. The experiment is only mooted so far, it is true, and it is hardly likely that the Oxford proctor's foot will be granted that privilege which has been denied to our national police. Undergraduates may well disclaim against so undignified a proceeding. The relations between proctors and their prey have ever been of that strained nature which characterizes the pursued and the pursuer; but were these "improvements" in the equipping of the foot actually to take place, these relations, it seems, would assume a yet more definite form, the old order of things would be reversed, and it would be the indiarubber shoe now which would have to flee the wrath of its would-be prey.

The question as to how much English women indulge in smoking has been a good deal before the public lately. And so diverse are the opinions on this subject that it is hard to come to any conclusion beyond the one readily arrived at, that if the fair sex do indulge to any great extent in this habit they certainly manage to keep it quiet amongst themselves, and the public only has a right to complain in so far as its feelings are obtrusively outraged. So far, in England this has not been the case. But an amusing exception to this state of things we have just met with in the pages of a weekly contemporary. It appears that the writer was travelling on the underground railway, when a quiet-looking, and well-dressed lady was stopped from entering a certain compartment by the guard, who informed her that it was one reserved for smokers. "That is what I want," replied the lady. "I want to smoke; don't let any men get in."

